# Socioeconomic status and prescribing for schizophrenia: analysis of 3200 cases from the Glasgow Psychosis Clinical Information System (PsyCIS)

**DOI:** 10.1192/pb.bp.112.042143

**Published:** 2014-04

**Authors:** Daniel J. Martin, John Park, Julie Langan, Moira Connolly, Daniel J. Smith, Mark Taylor

**Affiliations:** 1 University of Glasgow; 2 NHS Greater Glasgow and Clyde; 3 Royal Edinburgh Hospital

## Abstract

**Aims and method** To investigate whether socioeconomic status influenced rates of depot medication prescribing, polypharmacy (more than two psychotropic medications), newer (second-generation) antipsychotic prescribing and clozapine therapy. Postcodes, Scottish Index of Multiple Deprivation (SIMD) categories and current medication status were ascertained. Patients in the most deprived SIMD groups (8-10 combined) were compared with those in the most affluent SIMD groups (1-3 combined).

**Results** Overall, 3200 patients with ICD-10 schizophrenia were identified. No clear relationship between socioeconomic status and any of the four prescribing areas was identified, although rates of depot medication use in deprived areas were slightly higher.

**Clinical implications** Contrary to our hypothesis, there was no evidence that patients with schizophrenia within NHS Greater Glasgow and Clyde who live in more deprived communities had different prescribing experiences from patients living in more affluent areas.

Psychotic disorders are a complex and significant public health problem, with heavy personal, social and health-related costs.^1^ In addition to high rates of functional impairment, disability and reduced quality of life, patients with psychosis often have comorbid substance misuse and frequent side-effects from their medication.^1^ Despite extensive investment and highly developed community and in-patient services, patients with psychosis also suffer disproportionate levels of social isolation and socioeconomic deprivation.^1^ Studies of out-patient populations with schizophrenia indicate that these individuals are significantly more likely to be unemployed, have no formal qualifications and live in group homes.^2^ There have been reports that socioeconomic status (SES) can influence prescribing for schizophrenia. In a survey of 1342 physicians in Germany, Franz and colleagues^3^ found that non-adherent patients with schizophrenia with low SES were four times more likely to receive conventional depot or long-acting injections of antipsychotics than non-adherent patients of high SES. Conversely, high-SES non-adherent patients were more likely to be prescribed oral and long-acting depot second-generation antipsychotics.^3^

Greater Glasgow and Clyde Health Board has a higher concentration of deprived areas than the rest of Scotland, with 43.5% of the population belonging to the most deprived 15% of the Scottish population.^4^ NHS Greater Glasgow and Clyde has developed a comprehensive case register of patients with psychosis treated in secondary care over the past decade, the Psychosis Clinical Information System (PsyCIS),^5^ with details of over 7000 individuals included. The PsyCIS database facilitates clinical governance and research studies. In this report, we aimed to investigate the impact of SES as defined by the Scottish Index of Multiple Deprivation (SIMD) category^4^ on prescribing for patients with schizophrenia in Glasgow. Specifically, we aimed to determine how SIMD category influenced rates of: prescription of depot medication, polypharmacy (more than two psychotropic medications), prescription of newer second-generation antipsychotics and prescription of clozapine.

We hypothesised that patients with schizophrenia living in areas of high deprivation (SIMD 8-10 combined), relative to patients living in areas of high affluence (SIMD 1-3 combined), would have higher rates of prescribed depot medications and polypharmacy and lower rates of second-generation antipsychotic and clozapine prescribing. Although each individual patient may benefit from a number of different medications, the prescription of second-generation antipsychotic medication and clozapine was felt to be a marker of good care. The reasons for this include less extrapyramidal side-effects and that less monitoring is required with second-generation antipsychotics. Higher prescription rates of clozapine were thought to represent a pragmatic and systematic approach to severe and enduring mental illness within a patient group given that clozapine remains the only medication currently licensed for the management of treatment-resistant schizophrenia.

## Method

The PsyCIS register consists of details of adult (aged 18-65 years) patients in the NHS Greater Glasgow and Clyde Health Board area in contact with community-based mental health services, with an ICD-10 diagnosis of F20-29, F30-31, F32.3, F33.3 F06.0-06.2, F06.30-06.31 and F1(x) with psychotic symptoms, diagnosed by a consultant psychiatrist using ICD-10 criteria.^5^ A retrospective medical case-note audit across Greater Glasgow and Clyde (population approximately 1 million people) was initially undertaken to record relevant clinical and sociodemographic data on patients with a diagnosis code from those noted. Data were collected over a 42-month period, from February 2002 to August 2005. Over 8000 case notes were audited in total. Since September 2005 any patients in contact with community services with any of the noted diagnoses have continued to be registered on the system using the same methodology.

Where there was uncertainty over the primary diagnostic coding, case notes were reviewed by the research team in consultation with the local consultant psychiatrist and a clinical consensus diagnostic coding was applied. Annual update information is provided by psychiatrists involved in the direct care of the patient and includes updates on clinical status, treatment and sociodemographic circumstances. The process of annual review also enabled checking of the accuracy of information such as postcode and current medication. Local clinicians have a two-way relationship with the PsyCIS team, which facilitates the return to consultants of clinically relevant information at an individual case-load level. This study of prescribing practice was approved by the West of Scotland NHS Research Ethics Committee.

Overall, 3200 patients with ICD-10-diagnosed schizophrenia were identified as having been on the PsyCIS database between February 2002 and August 2005. Each of these patients was allocated to a SIMD category based on their postcode (1 = most affluent, 10 = most deprived). The index combines information from seven domains which carry different weightings, as follows: current income (28%), employment (28%), health (14%), education (14%), geographic access to services (9%), crime (5%), housing (2%).^6^

The most recently recorded medication information on the PsyCIS system was extracted for each patient along with information on gender and length of contact with psychiatric services; this was then analysed further. A descriptive analysis was carried out comparing prescribing rates of depot medication, polypharmacy, atypical antipsychotics and clozapine with SIMD category. Chi-squared tests comparing the most affluent group (SIMD 1-3 inclusive) with the most deprived group (SIMD 8-10 inclusive) were then carried out for each of the four prescribing areas.

## Results

Of the 3200 patients with ICD-10-diagnosed schizophrenia identified, 69.1% were male. The mean number of years in contact with services for men was 18.9 (s.d. = 12.1), and for women 21.6 (s.d. = 13.8) (*P* = <0.0001, 95% CI –3.580 to –1.6795); confidence intervals reflect differences in the mean ages between men and women. Almost half the patients (46%, *n* = 1656) were within the most deprived group, compared with only 9% (*n* = 335) from the most affluent group.

There were no significant differences for gender and age distribution between the affluent and deprived groups. In the affluent group 31.9% were female (mean age 50.2 years) and in the deprived group 29.5% were female (mean age 48.1 years). The mean age of men in the affluent group was 50.2 *v*. 48.1 years in the deprived group.

### Depot medication prescribing

The overall rate of depot long-acting antipsychotic prescription across both groups was 29.3% (range 22-34). Although not statistically significant (31% *v*. 26%; odds ratio (OR) = 1.3, 95% CI 0.98-1.67, *P* = 0.07), there was a trend towards greater use of depot medications in patients of lower SES.

### Polypharmacy

The overall rate of polypharmacy, defined as the prescription of more than two psychotropic medications, across both groups was 16% (range 11-18). Very similar rates of polypharmacy were seen in the affluent and deprived groups (17% in the deprived group *v*. 16% in the affluent group; OR = 0.92, 95% CI 0.67-1.26, *P* = 0.59). Furthermore, 11.81% of the total number of patients in the PsyCIS register were prescribed more than one antipsychotic medication.

### Second-generation antipsychotics

A comparison of affluent and deprived groups found similar rates of second-generation antipsychotic prescription (49% in the deprived group *v*. 47% in the affluent group; OR = 1.06, 95% CI 0.84-1.34, *P* = 0.63).

### Clozapine prescription

A comparison of affluent and deprived groups showed very similar rates of clozapine prescribing, with 17% of the deprived group being prescribed the drug compared with 19% in the affluent group (OR = 0.87, 95% CI 0.64-1.19, *P* = 0.39). Although similar rates of clozapine prescribing were found between the different socioeconomic groups, SIMD category 1 had a low rate of clozapine prescription (11% of the group).

## Discussion

**Fig 1:**
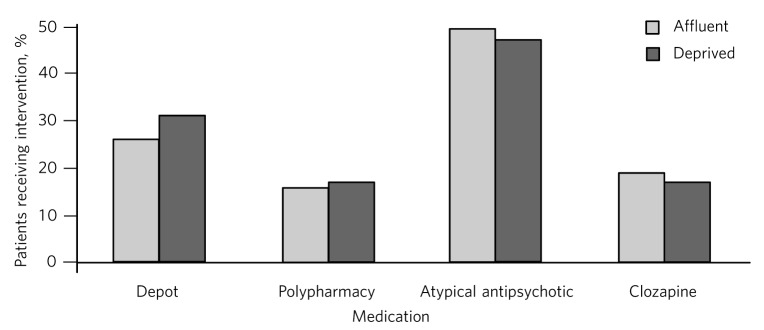
Prescribing practices in schizophrenia: affluent *v*. deprived groups.

A rather striking finding from this study is the very high rates of social and economic deprivation seen within this cohort. However, overall we found no clear relationship between SIMD category and any of the four prescribing areas (Fig. 1). One possible explanation for this apparently consistent prescribing approach across Glasgow might be the strong local continuing professional development and peer review mechanisms in place. Another possible explanation for this encouraging finding could be the use of the PsyCIS case register itself, which provides regular updates and feedback to local psychiatrists about the clinical, social and demographic features of their patients and therefore allows regular reflection on and subsequent improvements in prescribing through raised awareness and reflective practice.

The differences between our findings and those of Franz and colleagues^3^ are interesting. Franz *et al* found that low medication adherence was associated with an increase in selections of depot medication, and high-status non-adherent patients tended to receive atypical oral and atypical depot antipsychotics. They also found that non-adherent patients of low SES were mostly prescribed conventional and atypical depot antipsychotics. Patients who had difficulty with adherence and were of lower SES received first-generation injectable antipsychotics four times as often as non-adherent, high-SES patients. Possible explanations for the differences in findings are numerous, although different methodology should be highlighted. Franz *et al*’s study was based on reports from fictional vignettes as opposed to retrospective and ongoing analysis of case notes and electronic records in our study. The clear local prescribing protocols and guidelines within NHS Greater Glasgow and Clyde alongside strong support from pharmacy services may also partially explain the differences in findings.

One possible reason for the trend (albeit not statistically significant) towards greater use of depot medications in patients of lower SES may be the phenomenon of ‘social drift’, whereby severe psychotic disorders result in a lowering of SES. Severe psychosis is also likely to be associated with reduced medication adherence and behavioural disturbance, both of which may be associated with increased rates of depot medication.

Polypharmacy was defined in the PsyCIS cohort as the prescription of two or more classes of psychotropic medication. An Australian national survey of self-reported medication use in 1825 participants with psychotic illness reported that 69% of people on psychotropic medication had been using more than one psychotropic medication in the month prior to interview.^7^ The study also reported that 24.9% of patients with a psychotic illness were using more than one antipsychotic medication, compared with 11.81% of patients in the PsyCIS group who were prescribed more than one antipsychotic medication. This finding further underlines the reduced rate of polypharmacy within the PsyCIS cohort.

Previous studies have found rates of polypharmacy of up to 90% in patients with schizophrenia,^8,9^ albeit one of the studies^8^ was carried out in an in-patient setting, which may have resulted in increased rates of polypharmacy. A large American study of 13 079 visits to office-based psychiatrists indicated a rise in psychotropic polypharmacy (defined as the prescription of more than two psychotropic drugs), from 16.9% to 33.2% between 1996-1997 and 2005-2006.^10^ The studies have used different methodologies, were carried out in different settings and defined polypharmacy differently. It is still possible to make a broad comparison, with the rates of psychotropic polypharmacy in our study being low. The prescription of more than two psychotropic medication classes was chosen to define polypharmacy in this study given the presence of the American study which was robust and thorough. The relatively low rate of polypharmacy in our sample may be associated with clear clinical guidelines,^9^ increased attention in the literature, improved multidisciplinary input (e.g. from pharmacy) and improved reporting of polypharmacy by medical and nursing staff. It is also worth noting that PsyCIS highlights prescribing and other management strategies to psychiatrists working locally.

Rates of second-generation antipsychotic prescribing were also similar between different socioeconomic groups. Their widespread use may be due to the reduction in the costs and improved availability of these medications as well as clear guidelines on the use of atypical antipsychotic drugs for schizophrenia. There are reports of higher rates of second-generation or atypical antipsychotic prescribing within an Australian population. In a 2007 study of 2365 out-patients with schizophrenia which had similar methodology to our study, Wheeler reported that 81% of patients were prescribed an atypical antipsychotic.^2^ Evidence emerging since 2007 and subsequent increased awareness relating to the metabolic side-effects of these drugs may partly explain the differences in rates noted between Glasgow and Australia.

A comparison of affluent and deprived groups showed very similar rates of clozapine prescribing, with 17% of the deprived group being prescribed the drug compared with 19% in the affluent group. A possible reason for low prescription rates of clozapine in the SIMD 1 group alone (11%) could be the fact that individuals who have responded to initial treatments may have been able to retain higher SES. Chaotic social circumstances and high rates of comorbid substance misuse are associated with low SES, and are also relative contraindications for clozapine, given the regular monitoring and follow-up required for patients on the drug. Furthermore, treatment resistance to clozapine is likely to be associated with a worsening socioeconomic decline seen in schizophrenia. Investment and improvements in community psychiatric services throughout Glasgow in the past 10 years may have facilitated better patient engagement and enabled more robust monitoring of patients on clozapine, therefore increasing rates of prescription across the socioeconomic groups. In their 2000 study of in-patients, Taylor and colleagues found an overall clozapine prescription rate of 23%.^11^ It is encouraging that in Glasgow clozapine prescription rates are similar, although the PsyCIS database is based on out-patient data. The higher rate in Taylor and colleagues’ study may be due to in-patients having a greater likelihood of more severe or poorly controlled illness.

### Strengths and limitations

Strengths of the study include the large and comprehensive nature of the PsyCIS database, which is representative and prospective in design. Regular checks of data accuracy are carried out by the senior medical practitioners involved in case management, therefore improving the validity of recorded diagnoses, clinical and sociodemographic circumstances. Regular review also ensures up-to-date information is entered into the database.

Limitations include possible inaccuracies in data recording or reporting, although the frequent re-checking of notes and other sources of clinical information does reduce this possibility. Misdiagnosis is also a consideration, although again this is probably reduced by the use of consultant psychiatrist-based diagnoses and the checking of diagnoses against ICD-10 criteria.

Another possible limitation of the study is the exclusion of patients managed solely in primary care. This is inherent to the design of the study and would be difficult to improve on without a significant linkage of the PsyCIS database and primary care records. Although some patients with psychosis are able to live independently in the community without input from secondary services, further study is required to ascertain numbers of patients. Furthermore, PsyCIS also excludes those under 16 and over 65 years old not seen by general adult community services, in addition to those whose psychotic illness is managed exclusively in addictions psychiatry, old age psychiatry or learning disability services. These numbers are likely to be relatively small and the vast majority of patients of working age who have psychotic illness are managed by adult general psychiatric services. It should also be noted that using SIMD categories as a proxy measure of deprivation introduces limitations into the study, too. Postcode sectors that are not internally homogeneous may contain varying levels of deprivation.

Perhaps surprisingly, there are relatively few reports in the literature on the impact of SES on prescribing in schizophrenia. Our findings contrast with Franz and colleagues’ recent survey which indicated that low SES was associated with increased rates of depot prescription and reduced rates of atypical antipsychotic use. Franz’s work was, however, based on the responses of physicians rather than objective measures of prescribing, as in our study.
